# Trend in Detection of Anthocyanins from Fresh Fruits and the Influence of Some Factors on Their Stability Impacting Human Health: Kinetic Study Assisted by UV–Vis Spectrophotometry

**DOI:** 10.3390/antiox14020227

**Published:** 2025-02-17

**Authors:** Cătălina Ionescu, Adriana Samide, Cristian Tigae

**Affiliations:** Department of Chemistry, Faculty of Sciences, University of Craiova, Calea Bucuresti, 107i, 200144 Craiova, Romania; cristian.tigae@edu.ucv.ro

**Keywords:** anthocyanins, antioxidants, pH differential method, total anthocyanin content, kinetic study, degradation mechanism

## Abstract

Anthocyanins (ANTHs) are polyphenolic compounds with health promoting properties, being known for their strong antioxidant effects as well as for their antimicrobial properties, obesity and cardiovascular disease prevention, and anticarcinogenic activity. Being main dietary components, it is important to know the content of anthocyanins in various dietary sources and their stability in time. The total anthocyanin content (TAC) of various fresh fruits has been spectrophotometrically determined using the pH differential method. The results showed that in the analyzed samples, the TAC increased in the order: blackcurrants > blackberries > blueberries > raspberries > strawberries > plums. The degradation degree of anthocyanins extracted from blueberries (BBEs) in an ethanol/water solution in four experimental conditions was studied. Kinetic studies have been approached, fitting the experimental data recorded by UV–Vis spectrophotometric analysis in agreement with some kinetic models verified for the ANTH degradation reaction. Therefore, zero-order kinetics for BBE extract degradation exposed to sunlight were identified, while for the other storage conditions (shadow, dark, cold), the first-order kinetics were respected. The results indicate that the stability decreased as follows: (ANTH stability)_sunlight test_ << (ANTH stability)_shadow test_ ≈ (ANTH stability)_dark test_ < (ANTH stability)_cold test_. A mechanism for BBE anthocyanin degradation was proposed and the impact on human health of the degradation products is discussed.

## 1. Introduction

Anthocyanins or anthocyans are naturally occurring, water-soluble compounds belonging to the class of flavonoids, which are polyphenolic compounds synthesized in plants as secondary metabolites that give wonderful colors to plant fruits and leaves. Known to possess health promoting qualities, interest has been devoted to determine the dietary anthocyanin content in various plant-derived foods.

### 1.1. Chemical Structure

**Scheme 1:**
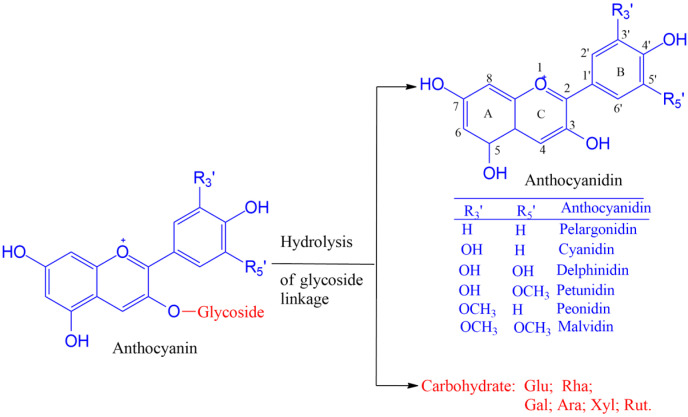
General anthocyanin structure and hydrolysis reaction.

Anthocyanins are glycosylated anthocyanidins, so their structure can be divided into two parts (that can be released by hydrolysis—Scheme 1) [1]. An anthocyanidin (aglycone) part is represented by the polyphenolic scaffold, with a flavonoid structure. All flavonoids share a common flavylium ion skeleton, having a C6-C3-C6 structure, which designates the number of carbon atoms in the three rings that form its structure: ring A, ring C, and ring B, respectively [2,3]. More than 30 anthocyanidins have been identified in nature, 6 of them being present the most often: pelargonidin, cyanidin, peonidin, delphinidin, petunidin, and malvidin. A carbohydrate part is represented mainly by glucose (glu) and rhamnose (rha), but galactose (gal), arabinose (ara), xylose (xyl), and rutinose (rut) may also be present [3]. The sugar can be free or acylated with various acids: (a) aliphatic acids—malonic, succinic, malic, and acetic acid; or (b) cinnamic acids—p-coumaric, ferulic, and sinapic acid [4]. Anthocyanins are most often 3*O*-glucosides of anthocyanidins.

The great variety of anthocyanins comes from the different combinations of anthocyanidins (as such or in methylated form) with various carbohydrates (in free or acylated form). Thus, anthocyanins group together more than 600 compounds identified in plants. However, the most widely identified anthocyanin in dietary sources and therefore the most studied compound is cyanidin-3-glucoside [4].

### 1.2. Anthocyanin Occurrence in Nature

Anthocyanins are present in plant tissues (leaves, stems, roots, flowers, and fruits) [5]. They are natural dyes and their color ranges from orange, red, and purple to blue and even black (the term “anthocyanin” derives from two Greek words: ἄνθος (anthos), meaning “flower” and κυάνεος/κυανοῦς kyaneos/kyanous meaning “dark blue”) [3]. Anthocyanins have traditionally been used as natural food dyes.

### 1.3. Health Benefits

Due to their potential health benefits, the interest in anthocyanin rich-diets has intensified in the last 20 years [6]. Anthocyanins are polyphenolic compounds, recognized for their health benefits like their antioxidant properties, which are known to be important characteristics of polyphenolic compounds, beneficial for the prevention of chronic diseases [7].

Being able to neutralize free radicals, anthocyanins possess high antioxidant activities and decrease oxidative stress in cells, having protective effects at the molecular and cellular levels. They are able to protect the structural integrity of biomolecules and of cells, sustaining health and body repair processes. Besides the antioxidant activity, anti-inflammatory and antimicrobial properties of anthocyanins have also been reported [8,9,10]. Other health benefits include hepatoprotective and nephroprotective properties, obesity and cardiovascular disease prevention, anticarcinogenic activity, neuronal, and vision improvement, properties that have been reviewed in the literature [3,11,12,13,14,15,16,17].

### 1.4. Anthocyanins Stability and Kinetics of Degradation

Anthocyanins are valuable dietary components; therefore, it is important to have information regarding anthocyanin behavior during food processing and storage. The stability of anthocyanins and their degradation kinetics have been investigated in various conditions. Factors like the influence of pH, co-pigmentation effect, metallic interaction, self-association, intramolecular and intermolecular co-pigmentation, temperature, oxygen, ascorbic acid, light, sulfites, enzymes, and effect of encapsulation have been investigated and reviewed [17]. The most studied factors have been pH and temperature. The temperature effect has been intensively investigated, as most food processing involves heating. Studies indicate that low pH and low temperature are the best conditions for blueberry anthocyanin preservation [18].

The reaction kinetics of anthocyananin degradation conditions fitted zero- and first-order kinetics. Most studies have reported first-order kinetic degradation of anthocyanins from various vegetal sources under isothermal heating conditions [19,20,21,22,23,24], but zero-order kinetics have also been mentioned. When blueberry puree was subjected to hydrothermal processing, first-order reaction kinetics for anthocyanin degradation were reported, while polymeric color formation followed zero-order kinetics [25]. A recent study showed that under mixed sonophotolysis and sonolysis treatment, the degradation of cyanidin-3-O-glucoside underwent zero-order kinetics, while under photolysis, the main degradation kinetic was first-order [26]. Studies of the behavior of total anthocyanins as well as of individual anthocyanins from blueberry puree under low-oxygen and conventional pulping combined with high hydrostatic pressure indicated that the degradation kinetics during storage at 4 °C of individual anthocyanins as well as those of the total anthocyanins followed the first-order kinetic model, with two exceptions: delphinidin-3-glucoside and peonidin-3-galactoside, which followed the zero-order kinetic model.

Oxygen presence is a degradation factor for anthocyanins. Oxidation reactions catalyzed by polyphenol oxidase (PPO) and peroxidase (POD) are completely inhibited after sample exposure at temperatures above 80 °C, as enzyme denaturation takes place [25]. In a study aimed to determine the effect of catechin co-pigment on the stability of anthocyanins in *Clitoriat ernatea* flower extract, it was reported that anthocyanin degradation followed the zero-order kinetics at three temperatures (28, 60, and 90 °C) [27].

Most of these studies investigated the behavior of ANTHs in aqueous solutions, for the purpose of studying ANTH stability during food processing and storage, which takes place in an aqueous medium in the food matrix, the most studied factor being temperature.

Possible applications of ANTHs in the food industry involve their usage as natural coloring agents and as nutraceutical agents due to their antioxidant properties. ANTHs are water soluble, but they also dissolve in alcohols. Anthocyanin aglycones are more soluble in alcohol solutions than anthocyanins [15]. Less information is known on ANTH extraction and stability in alcoholic solutions. Some studies in this field have been conducted [28,29,30], but their number is very limited. These indicate that ANTH degradation kinetics in ethanol solutions are of the apparent first-order [28], second order [29], or respect the Weibull model [30] when subjected to thermal or light degradation.

In some of these studies, pure anthocyanins were used or ANTHs were subjected to artificial light irradiation. ANTH behavior and degradation kinetics are different when the chemical and physical environment changes. Pure ANTHs have a different kinetic behavior from ANTHs present in a mixture with other components present in the food matrix or as a function of lightning irradiation used in the experiment.

We found it of interest to investigate the behavior of ethanolic ANTH extracts under natural environmental conditions, both for alcoholic beverages and for ANTH extracts with nutraceutical applications, for the purpose of finding the best storage and packaging conditions, as little information is available on this subject.

This study had as its purpose the determination of the total monomeric anthocyanin content in various fruits and the investigation of their stability under the usual conditions during storage in ethanol/water mixtures using UV–Vis spectrophotometry.

## 2. Materials and Methods

### 2.1. Materials

#### 2.1.1. Samples

Six fruit types available at the local stores (blackcurrants, blackberries, blueberries, raspberries, strawberries, and plums) were used for the analysis.

#### 2.1.2. Reagents and Solutions

All reagents were purchased from Merck (Darmstadt, Germany). The extraction solvent was a mixture of ethanol/water 70/30 *v*/*v*. Two solutions were used for the analysis of the total anthocyanin content (TAC): (a) a potassium chloride/hydrochloric acid solution (0.025 mol L^−1^ KCl/HCl, pH 1.0), and (b) a sodium acetate solution/hydrochloric acid solution (0.4 mol L^−1^ CH_3_COONa/HCl, pH 4.5) [31].

#### 2.1.3. Apparatus

The spectrophotometric measurements were performed with a Varian Cary 50 UV–Vis spectrophotometer (Varian Inc., Mulgrave, VIC, Australia), with CaryWinUV software, version 3.0(303) using quartz and plastic cuvettes for a spectrophotometer with a 1 cm path length. Fruit samples and reagents were weighed using an Adam PW124 Lab Balance (Kingston, UK). A Consort C533 pH-meter (Consort nv Parklaan 36 B2300, Turnhout, Belgium) was used for the pH measurements. Fruit extracts were centrifuged using a Sigma 2-16K centrifuge (Osterode, Germany).

### 2.2. Methods

#### 2.2.1. Determination of Total Anthocyanin Content (TAC) in Fresh Fruits

##### Extract Preparation

A total of 10 g of each fruit was weighed and homogenized in a blender for 5 min with 50 mL of the ethanol/water extraction solvent. The obtained suspensions were centrifuged at 10,000 rpm for 10 min, and the supernatants were further analyzed.

##### Preparation of Test Solutions

For each determination, two test solutions were prepared: (a) 1 mL of extract + 4 mL of potassium chloride/hydrochloric acid solution (pH 1), and (b) 1 mL of extract + 4 mL of sodium acetate/hydrochloric acid solution, (pH 4.5). The pH differential method was used to determine the anthocyanin concentration in the extract solutions [31].

##### Calculations

The anthocyanin concentration (AC) in the extract solutions, expressed as cyanidin-3-glucoside (cyd-3-glu) equivalents (mg/L), were calculated using Equation (1), adapted from Liu et al. [18].

(1) $$AC\left(in\;extract\right)=\frac{5\times 10^{3}A\times M\times DF}{\varepsilon \times l}(mg/L)$$

where A is the absorbance calculated with Equation (2). The absorbance calculation was based on the pH differential method involving the spectrophotometric determination of the absorbance value at two wavelengths, 520 nm and 700 nm, respectively, for two distinctive pH (1 and 4.5 in this work).

(2) $$A=(A_{520}-A_{700}{)}_{pH=1}-(A_{520}-A_{700}{)}_{pH=4.5}$$

M = Molecular mass of cyanidin-3-glucoside = 449.2 g/mol;
DF = dilution factor;
l = pathlength in cm = 1 cm;
ε = extinction coefficient for cyd-3-glu (L·mol^–1^·cm^–1^) = 26,900 L·mol^−1^·cm^−1^;
AC (in extract) = A × 83.49 (mg cyd-3-glu/L solution).

Two stages (I and II) were used to calculate the TAC (fruit) as presented below.

As stated above, 60 mL of the ethanol–fruit mixture contained 10 g of fruit (noted further with m_f_) and therefore, if the concentration of anthocyanins in 1000 mL is AC in the 60 mL extract, respectively, in 10 g of fruit, it will be:

(3) $${AC}_{10\;g\;fruit}=\frac{6\times AC}{100}\;mg\;anthocyanins/m_{f}$$

Thus, for 100 g of fruit, it obtains:

(4) $${AC}_{100\;g\;fruits}=\frac{\frac{6\times AC}{100}\times 100}{m_{f}}\;mg\;anthocyanins/100\;g\;fruit$$

where m_f_ = 10 g fruit.

Consequently, the total anthocyanin content (TAC) in fruits was calculated with Equation (5):

(5) $$TAC{\left(fruit\right)}^{-}=\frac{6\times AC}{10}\;(\frac{mg\;cyd-3-glu}{100\;g\;fruit})$$

where AC = anthocyanin concentration (AC) in the extracts (mg cyd-3-glu/L solution).

For blackcurrants, blackberries, and blueberries, the results were multiplied with the dilution factor.

Statistical data. For each analysis, at least three individual determinations were realized. The data were expressed as mean and standard deviations (SD). The calculations and graphs were realized using Excel.

#### 2.2.2. Study of Blueberry (BBE) Anthocyanin Degradation

A total of 140 g of fresh blueberries was extracted with 350 mL of the ethanol/water 70/30 *v*/*v* mixture, which was used in order to obtain a diluted extract as follows: 27 mL of the initial extract was diluted with the ethanol/water 70/30 *v*/*v* mixture to a total of 81 mL, having a natural pH of 4.75. The extract was divided into 4 samples each, subjected to the fallowing storage conditions: (1) no light, 4 °C (cooling to refrigerator), further named the cold test; (2) dark, indoor conditions, room temperature, when contact with the light was interrupted by isolating the sample in aluminum foil (26 ± 2 °C), denoted as the dark test; (3) shadow, indoor conditions, room temperature, sample stored on a table in natural conditions, but not in incident sunlight (27 ± 5 °C), referred to below as the shadow test; (4) sunlight, indoor conditions, at room window, in incident sunlight (ranging from 36 ± 2 °C, day time to 25 ± 3 °C, night time), herein after referred to as the sunlight test. Every two days, aliquots were removed from each sample and two test solutions were prepared: (a) 1 mL of extract + 4 mL of potassium chloride/hydrochloric acid solution (pH 1), and (b) 1 mL of extract + 4 mL of sodium acetate/hydrochloric acid solution (pH 4.5). Absorbance was measured for each pH at 520 nm and at 700 nm. Absorbance (A) was determined as previously described using Equation (2).

#### 2.2.3. Kinetic Approach-Theoretical Considerations

Different kinetic models were applied to quantify the degradation processes of anthocyanins from blueberry extracts (BBE).

The experimental data obtained by spectrophotometric analysis were fitted according to the kinetics of zero- and first-order reactions using the specific equations based on which the linear diagrams A = f(t) and ln(A_o_/A) = f(t), respectively, were designed.

A_o_ and A represent the absorbance values at the initial time (t = 0) and at well-determined moment t, every two days. Practically, the slopes of the straight lines represent the rate constants (k) of the BBE degradation reactions under the laboratory storage conditions.

To better explain the application of kinetics, it is necessary to show the equations that express the variation in the concentration of BBE anthocyanins, noted further as [ANTH], over time, for each kinetic. Equations (6) and (7) reveal the variation of ANTH concentration, in time, for the kinetics of the zero- (6) and first (7)-order reactions.

(6) $$-\frac{d\left[ANTH\right]}{dt}=k$$

(7) $$-\frac{d\left[ANTH\right]}{dt}=k\cdot [ANTH]$$

Since the absorbance is directly proportional to the concentration of ANTH, Equations (6) and (7) can be rewritten as (Equations (8) and (9)):

(8) $$-\frac{dA}{dt}=k$$

(9) $$-\frac{dA}{dt}=k\cdot A$$

Consequently, by integrating the last two expressions, the rate equations corresponding to the kinetics of zero- and first-order, respectively, the following reactions are obtained (Equations (10) and (11)).

(10) $$A=A_{o}-k\cdot t$$

(11) $$ln\frac{A_{o}}{A}=k\cdot t$$

where t represents the time (days) and k is the reaction rate constant.

Thus, for zero-order reactions, the absorbance must vary with the R-squared value on the chart (R^2^) close to unity, according to Equation (10). In contrast, for the first-order reaction, the absorbance must have an exponential drop, as Equation (12) describes.

(12) $$A=A_{o}e^{-kt}$$

Consequently: (i) for the reactions respecting a zero-order kinetics, by plotting A = f(t), a straight line will be obtained whose slope (dA/dt) is equal to the rate constant (k), and the intersection with the ordinate represents the initial absorbance (A_o_); (ii) for first-order kinetics, the graph ln(A_o_/A) = f(t) represents a straight line with a slope [$\frac{d(ln\frac{A_{o}}{A})}{dt}$] equal to k and simultaneously, A = f (t) must display an exponential decrease in absorbance over time. The best description of the kinetic model will be provided by the R^2^ value.

The half-life (t_1/2_) represents the reaction time when the ANHT initial absorbance has decreased by half. The half-life expressions deduced from Equations (10) and (11) are presented below, as follows: Relation (13) for the kinetics of the zero-order reaction and (14) for the first-order reaction, respectively.

(13) $$t_{1/2}=\frac{A_{o}}{2\cdot k}$$

(14) $$t_{1/2}=\frac{ln2}{k}$$

## 3. Results and Discussion

### 3.1. pH Differential Method Application

For each test solution, absorbances were spectrophotometrically determined at two wavelengths: 520 nm and 700 nm. For certain samples (blackcurrants, blackberries, blueberries), dilutions were realized in order to fit the method The TAC was determined using the pH differential method, which was based on the observation that anthocyanins change color when the pH is varied. When initially introduced by Sondheimer and Kertesz in 1948 [32], the method used 2 and 3.4 as the pH values, but since then, the method has been refined, and currently, the two measurements are realized at pH 1 and 4.5 [33,34,35,36,37].

**Figure 1:**
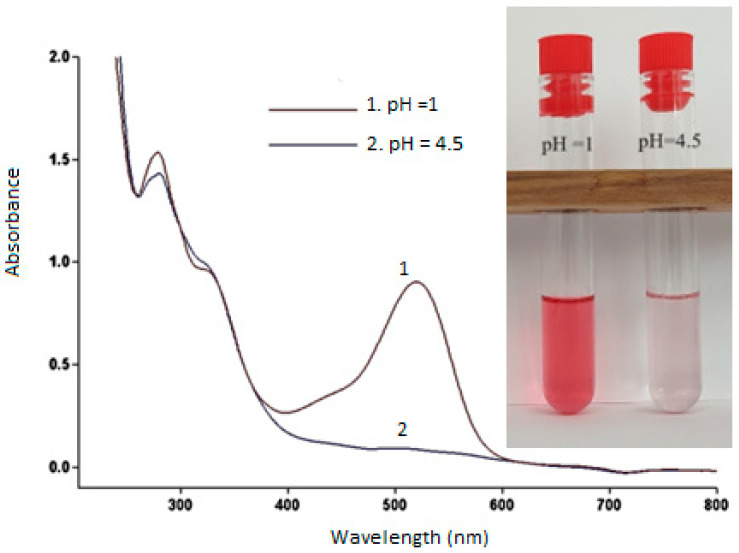
Absorption spectra of the ethanol/water blueberry extract at pH = 1 and pH = 4.5, inserting the two samples to visually detect the color change.

The plot inserted in Figure 1 shows that the color of the ethanol/water blueberry extract changed depending on the pH. The absorption spectra of the two samples recorded between 200 and 800 nm are displayed in Figure 1. An absorption maximum, at 520 nm, is highlighted on the spectrum obtained at pH = 1, while at pH = 4.5, this was not nuanced, with only an alteration in the baseline being recorded.

**Scheme 2:**
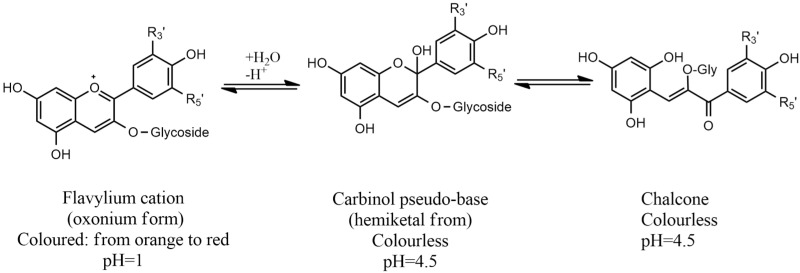
Change in anthocyanin structure from pH = 1 to pH = 4.5.

When the pH is varied, anthocyanins undergo a known reversible structural change at the level of ring C, based on literature data [33,34,38,39,40]. The anthocyanin structures are presented in Scheme 2:

(a) at pH = 1, anthocyanins are present as a colored oxonium form;
(b) at pH = 4.5, anthocyanins are present in a colorless hemiketal form [33,34,38,39].

At higher pH value levels, a quinoidal blue base is obtained [33,34,40].

Other colored substances or degraded anthocyanins attached to polymeric substances in the analyzed samples (well-known phenomenon for the tannins in wine) absorb color at both pH values (as could also be observed for our samples, Figure 1—test tube in the right side, pH = 4.5). Therefore, the anthocyanin content was calculated based on the difference in absorbance between the two pH values. For each pH value, absorbance was measured at 520 nm and at 700 nm.

### 3.2. Preliminary Analyses of the Samples

**Figure 2:**
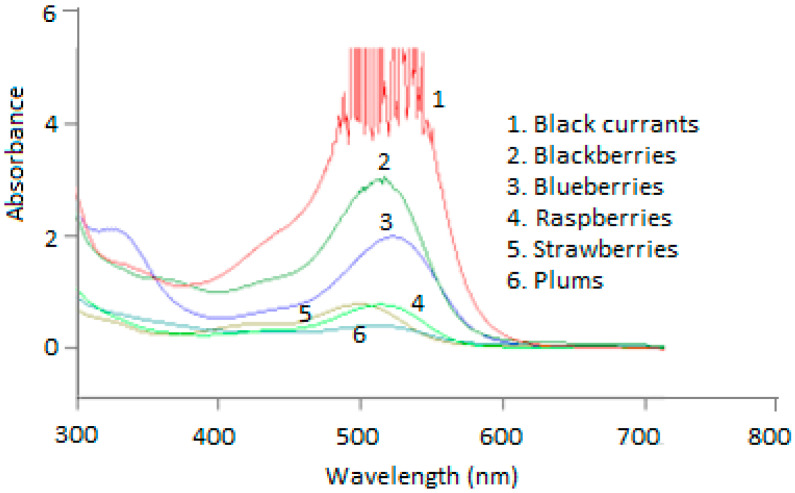
Absorption spectra of the extracts (without dilution) at pH = 1.

The absorbance spectra recorded between 300 and 800 nm are shown in Figure 2. It can be observed that for blackcurrants, blackberries, and blueberries, the absorbance maximum (λ = 520 nm) at pH = 1 was higher than that at 1.4, as requested by the method. Therefore, these samples were diluted as follows prior to the final analysis and data interpretation: blackcurrants (d = 10), blackberries (d = 5), and blueberries (d = 5). For the other fruits, the extracts could be analyzed directly after centrifugation.

### 3.3. TAC Determination

**Figure 3:**
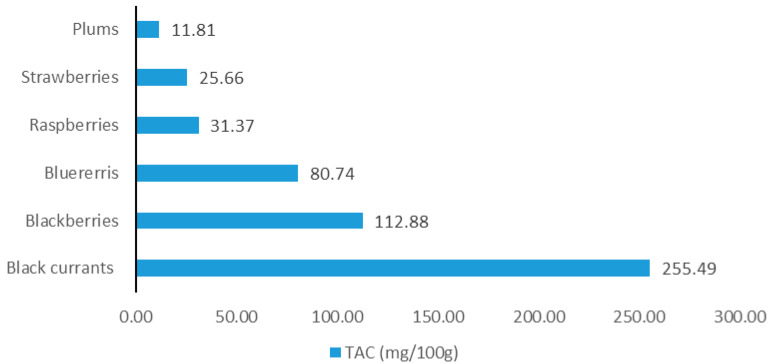
Total anthocyanin content, TAC (mg/100 fresh fruit) in the analyzed fruits.

The obtained experimental results are presented in Table 1. Starting from the read absorbances, using Equations (1), (2) and (5), the anthocyanin concentration (AC) in the obtained extracts as well as the total anthocyanin content (TAC) in fruits were calculated. TAC values, expressed as the means and standard deviations, represent the mg of cyanidin-3-glucoside/100 g fruit. The obtained results were in agreement with the data reported in the literature for the analyzed fruits, as shown in Table 1 and Figure 3.

### 3.4. Degradation Study of Blueberry (BBE) Anthocyanins

#### 3.4.1. Visual Assessment

**Figure 4:**
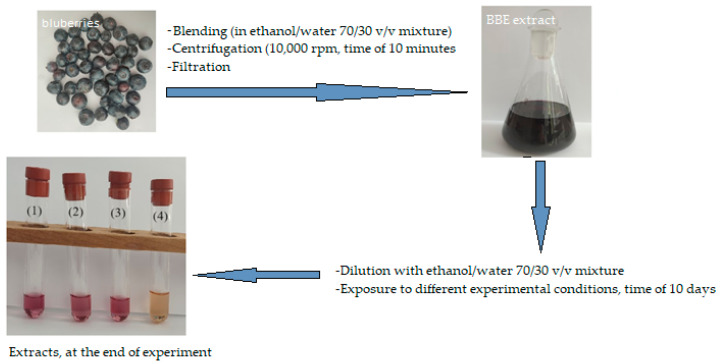
Description of experiment investigating the anthocyanin stability in BBE, in ethanol/water mixtures, for 10 days; experimental conditions of storage: (1) cold test; (2) dark test; (3) shadow test; (4) sunlight test.

In order to study the decomposition/degradation of anthocyanins, blueberries (BBE) represent a good choice due to their extract, which has a visually identifiable appearance, providing an accurate spectrophotometric signal at a wavelength λ = 520 nm (see Figure 1). Additionally, the fruits are easily procured, maintaining their freshness a certain time until processing, and during the investigation, the extract color changing was easily noticeable. The degradation of anthocyanins was performed in a water/ethanol solution, constituting a mixture that can be frequently found in cocktails and alcoholic drinks prepared from blueberry extracts. The blueberry extract was subjected to different indoor storage conditions denoted as the cold test, dark test, shadow test, and sunlight test (see Section 2.2.2 for details). A visual estimation of the degradation could be made based on the picture illustrated in Figure 4. It can be seen that the color change was much more obvious for the sunlight test compared with the other three samples. Apparently, during the 10 days, only the sunlight test affected the BBE extract.

#### 3.4.2. Calculation of the Degradation Degree of BBE Anthocyanins

It is difficult to draw any conclusions after only a simple visual evaluation. A clear and consistent quantification of the degradation of anthocyanins in blueberries can be achieved by computing the degradation degree and applying an appropriate kinetic model to determine the rate constant (k) of the degradation reaction and half-life of the concentration of anthocyanins. For this purpose, the absorbance values at wavelengths, λ = 520 nm and λ = 700 nm, were determined at pH 1 and 4.5, at a time of 10 days, under the storage conditions stated above. The calculated absorbance values, taking into consideration the standard deviation, are listed in Table 2.

**Figure 5:**
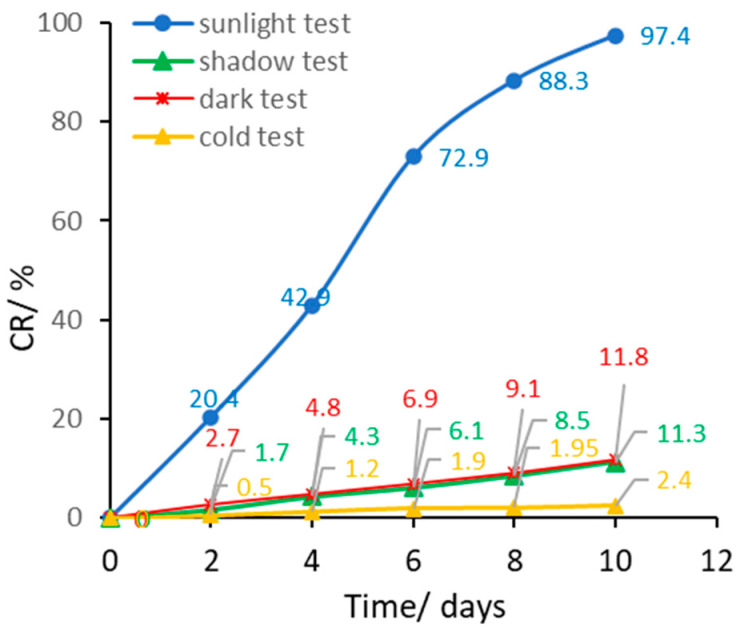
The degradation degree of BBE anthocyanins in the ethanol/water solution under the experimental conditions of storage.

The degradation degree of BBE active compounds that could be assimilated to the degree of color removal (CR%) was calculated using Equation (15), and is displayed in Figure 5 for each sample under the experimental storage conditions.

(15) $$CR\%=\frac{A_{o}-A}{A_{o}}\times 100$$

where A_o_ and A represent the absorbance values at the initial time and at a rigorously established moment t. The absorbance determination was performed every other day, at the same time of day.

As Figure 5 shows, the most pronounced decomposition (97.4%) after ten days was achieved in the case of the sunlight test, a similar conclusion to that drawn from Figure 4. The discoloration of the extract is obvious, which leads to a significant change in its characteristics. The BBE anthocyanins presented the highest stability in the cold test, the CR reached a low value of 2.4%, and the change in the extract appearance could not be visually detected in Figure 4.

In the case of the other two tests (shadow and dark), almost similar values of the BBE degradation degree (around 11%) were obtained, and an insignificant increase was noticed for the sample exposed in the dark. However, over a longer time, in the shade, more nuanced changes can take place compared with the ones highlighted in the dark, caused by the amplification of the effect of absorbed light. Visually, it is difficult to notice a change in the extract color and even more for an undiluted extract (Figure 4), where the color intensity is significantly higher, thus outlining in a debatable way, the preservation of freshness.

Consequently, even alcoholic extracts (beverages, essences, essential oils) should be kept at a temperature that does not exceed 18 ± 2 °C, and the validity time should involve the possibility of degradation of the active compounds.

### 3.5. Kinetic Approach

#### 3.5.1. Application of Zero-Order Reaction Kinetics

**Figure 6:**
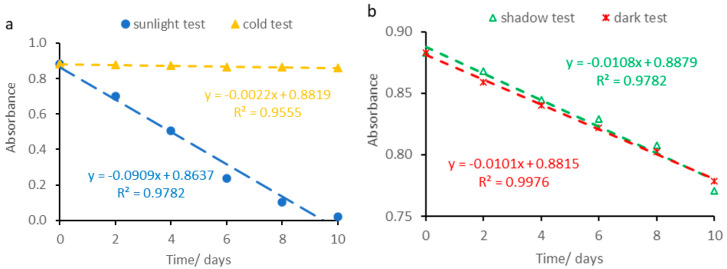
Verification of zero-order reaction kinetics for ANTH degradation from blueberry extracts exposed to different conditions of storage: (**a**) sunlight and cold tests; (**b**) shadow and dark tests.

Figure 6 presents the results obtained by applying the zero-order kinetic model for the BBE anthocyanin (ANTH) degradation reaction.

The ANTH degradation reaction rate constants were calculated by deriving the equations inserted in the graph from Figure 6. It was observed that the highest rate constant of 0.09 days^−1^ was assigned to the sunlight test, and the lowest to the cold test, reaching 0.0022 days^−1^ (Figure 6a). Thus, a behavior similar to that described in the previous paragraph can be highlighted.

For the shadow and dark tests, very close values were obtained, around 0.01 days^−1^, slightly higher for the one in the shade, which would not be in full agreement with their assigned degradation degrees. The comments will be detailed after checking the kinetics of the first-order reactions.

#### 3.5.2. Application of the First-Order Reaction Kinetics

**Figure 7:**
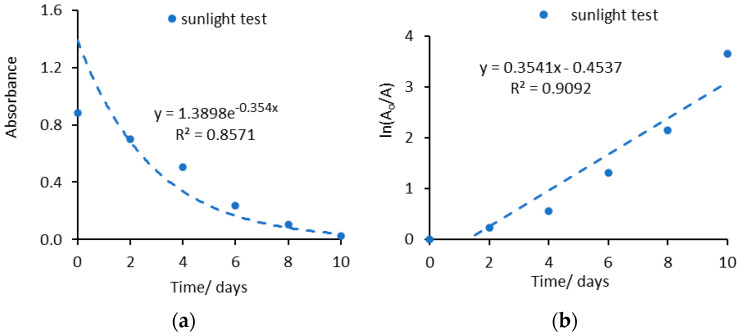
The first-order reaction kinetics for ANTH degradation for the sunlight-exposed BBE extract test: (**a**) the absorbance decrease trend over time; (**b**) reaction rate law verification.

Figure 7 shows that the model of the first-order kinetics for the sunlight test was inadequate, because the coefficient R^2^ had relatively small values for both the absorbance exponential decrease (0.8571) and the reaction rate law (0.9092). In this case, the ANHT degradation from BBE was definitely controlled by zero-order kinetics.

**Figure 8:**
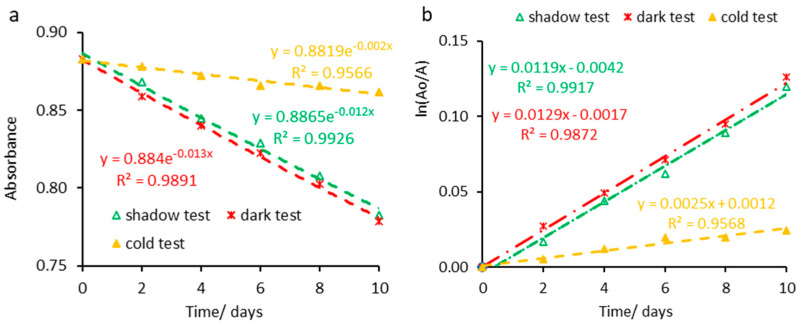
The first-order reaction kinetics for ANTH degradation for the shadow, dark, and cold tests: (**a**) the absorbance decrease trend over time; (**b**) reaction rate equation verification.

As shown in Figure 8, for the other three tests (shadow, dark, and cold), by fitting the experimental data according to the kinetic model related to the first-order reactions and applying both Equation (11) (Figure 8b) and Equation (12) (Figure 8a), respectively, the following statements can be made: (i) the R^2^ coefficients reached comparable values to those obtained from zero-order kinetics; (ii) however, the values of the initial absorbances determined from the equations inserted in Figure 6 and Figure 8a, like the y(x = 0) value, were closer to the experimental one (0.8829) when the fitting was carried out according to the kinetics of the first-order reactions.

On the other hand, in the case of the sunlight test, the calculated absorbance value of 1.3898 from Figure 7a (first-order kinetics) was much higher than the experimental one (0.8829), in contrast to the absorbance calculated from the zero-order kinetics, which reached a value of 0.8837, very close to the experimental one.

#### 3.5.3. Comparative Discussions Based on the Two Approached Kinetic Models

The kinetic parameters obtained by fitting the experimental data according to the zero- and first-order kinetics are comparatively presented in Table 3.

The degradation reaction of anthocyanins proceeded according to zero-order kinetics in the case of the blueberry extract’s exposure to sunlight, and respected the first-order kinetics for the other storage conditions (shadow, dark, and cold).

The half-life constitutes another parameter based on which ANHT stability can be evaluated. The higher the half-life, the more the degradation takes place with a lower reaction rate. The half-life was determined with Equation (13) for the zero-order kinetics and Equation (14) for the first-order kinetics (Table 3). Analyzing the data from Table 3, it can be seen that for the shadow and dark tests, inadvertent data were obtained with those computed for the ANTH degradation degree assimilated to color removal (CR, Figure 5), suggesting that it slightly reversed their stability. Thus, CR (shadow test of 11.3) < CR (dark test of 11.8), indicating slower degradation in the shade, which was not in good agreement with the information provided from the zero-order kinetics, where k (shadow test) > k (dark test) and t_1/2_ (shadow test) < t_1/2_ (dark test), meaning a change in the initial ANTH stability order. These inaccuracies were corrected by first-order kinetics, which provided data in full agreement (k and t_1/2_) with the degradation degree (CR) for the two tests in the shadow and dark.

For the cold test, from the first-order kinetics, a slightly higher value of R^2^ (Equation (11) and a calculated absorbance very close to the experimental one compared with those provided by the zero-order kinetics were noted. Consequently, the synergistic action of light and temperature favored the decomposition/degradation of anthocyanins, in a relatively short time, with a rate significantly higher than that of the samples for which contact with light was restricted, especially the one tested in the cold.

Thus, the stability of anthocyanins from the BBE extracts, depending on the storage conditions, varied as follows:

(ANTH stability)_sunlight test_ << (ANTH stability)_shadow test_ ≈ (ANTH stability)_dark test_ < (ANTH stability)_cold test_

#### 3.5.4. Deficiencies That Were Taken into Account for the Kinetic Approach

(1) The alcohol/water blueberry extract is a multicomponent mixture, thus creating the possibility of the simultaneous degradation of anthocyanins and other compounds; however, it is unlikely that other compounds and/or decomposition products interfere with the anthocyanins at the same wavelength, λ = 520 nm;

(2) The samples were exposed in an ambient environment (except the cold test), where temperature variations between night and day can influence the degradation reaction rate; however, we opted for a natural environment as close as possible to that of storage in warehouses, stores, or at home;

(3) Research presents certain risks that can be mitigated by handling, analyzing, and accurately processing the experimental data;

(4) For samples exposed to shadow, dark, and cold, the absorbance variations were small, which could have led to inadvertent and incoherent results; for this reason, a slight deviation from the first-order kinetics can occur;

(5) For accuracy, we considered the possibility of a slight deviation from the first-order kinetics, and consequently, in this study, we assumed a pseudo-first-order kinetics.

Based on the arguments stated above, the order of the degradation reaction of anthocyanins depending on the BBE extract storage conditions are shown in Table 4.

#### 3.5.5. Chemical Kinetics Versus Kinetic Studies in Living Organisms

In living organisms, the degradation of anthocyanins can proceed through a synergistic mechanism that consists, on the one hand, of the impact of environmental factors, such as gastric pH, temperature, and multiple interactions with other chemical compounds and enzymatic biocatalysis, on the other hand.

The biotransformation processes from ingestion to elimination are exhaustive-complicated involving the research and understanding of the way, rate, and degree of adsorption, the amount stored in the tissues and viscera, chemical and enzymatic degradation, the elimination rate over time of degradation products as well as the cumulative interactions and the sensitivity of the species that were subjected to an experiment.

Anthocyanins are relatively stable at acidic pH, but their accumulation and storage in tissues and organs over a long time can affect their integrity and stability. As a result, the appearance of some degradation compounds that are foreign to the biochemistry of the basic metabolism, superimposed with other disturbing causes, such as poor nutrition, stress, surgical interventions, chronic dysfunctions, pollution, drug consumption, generate the alteration of some cells or create an imbalance in the production of free radicals.

Certainly, the kinetic study in living organisms requires a different approach compared with chemical kinetics. Thus, the investigations were carried out over a long time involving intensive research on various species of animals, taking into account the multitude of internal and external factors that can alter the expected biological response.

The chemical kinetics performed on the simulated laboratory environments provided relevant results on the anthocyanin decomposition reaction rate under the influence of external factors, namely light and temperature, highlighting that the chemical stability is precarious and sometimes altered in an unexpected way. Thus, the chemical kinetics can be a starting point for more elaborate studies, indicating a possible decomposition/degradation reaction order of anthocyanins.

Certainly, the half-life values change under the conditions existing in living organisms, but the chemical kinetics reveal that there is also the possibility of an almost total decomposition over a relatively short time, which could change the reaction order from the first-order (softer conditions) to zero-order (more drastic conditions).

### 3.6. The Degradation of Anthocyanins

**Scheme 3:**
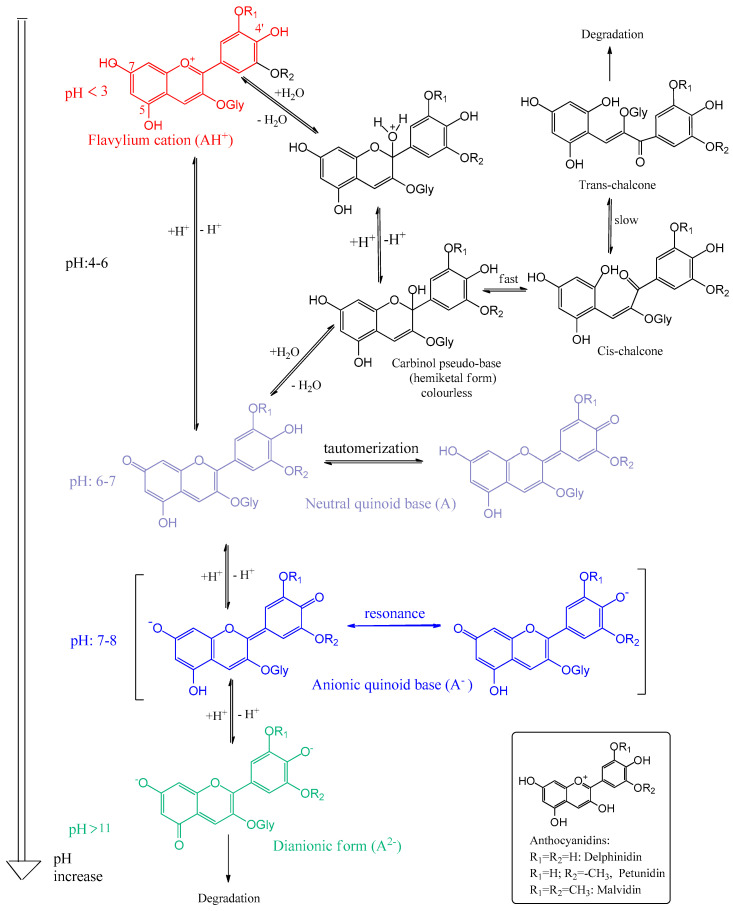
Cationic, neutral, and anionic forms of the 3 main anthocyanins found in blueberries at different pH values (derived from anthocyanidins: delphinidin, malvidin, and petunidin).

The anthocyanin structure is pH-dependent and undergoes deprotonation with increasing pH, generating various forms with specific colors and different stabilities. In Scheme 3, these equilibria are exemplified for the main anthocyanins found in blueberries. These fruits contain more than 15 anthocyanidins, but delphinidin, malvidin, and petunidin have been identified as their main components [48,49,50,51,52]. Both malvidin and petunidin are derived from delphinidin, malvidin being delphinid’s 3′,5′-dimethoxy derivative, while for petunidin, it is 5′-methoxydelphinidin. Anthocyanins possess three acidic phenolic groups, 7-OH, 4′-OH, and 5-OH, with -OH at C7 being the most acidic, followed by 4′-OH, and finally 5-OH [53]. The following forms are obtained, with increasing pH:

(1) In acidic conditions (pH ˂ 3), anthocyanins are found mainly in the form of a stable orange to red flavylium cation; in very acidic conditions (pH ˂ 1), the flavylium cation is the only species present in solution;
(2) At higher pH (6–7), the flavylium cation loses one proton (-OH at C7) and becomes a stable quinoid anhydrobase (violet). This form is in equilibrium with a less stable tautomer (C7-C4′ tautomerism);
(3) If the pH continues to increase (7–9), anthocyanins turn into an anionic quinone. Electrons delocalization over the three aromatic rings between C7 and C4′ allow for two resonance structures that stabilize the anion A^−^;
(4) At strong basic pH (˃11), an unstable dianionic form is obtained (blue-green color); this leads to degradation products through anionic chalcone species;
(5) Anthocyanins are present in water-containing solutions as a mixture of numerous structures, having cationic, neutral, and anionic forms upon the pH value of the solution. Our solutions had a measured pH value of 4.75, thus involving structures that are placed in the upper part of Scheme 3, and the degradation pathway of ANTH in the cold, dark, and shadow tests would probably undergo as described below.

At pH values between 4 and 6, four species co-exist (flavylium cation, quinoidal base, carbinol pseudo-base, chalcone), with the flavylium cation as the main component (Scheme 3) [46]. The carbinol-pseudo-base is obtained through the addition of water to the flavylium cation and can undergo fast transformation into the cis-chalcone, which can be slowly isomerized into the trans-chalcone [53]. This cis-trans isomerization process takes several hours in anthocyanins [54]. One important mechanistic finding is that the quinoidal base does not hydrate in an acidic medium, therefore, chalcones are formed at acidic pH starting from flavylium cations only [38]. Chalcones are obtained through the opening of the pyrylium ring C through an endothermic process, therefore, a temperature increase in anthocyanin solutions favors the preponderance of the chalcone form over the other three species (flavylium cation, quinoidal base, and carbinol pseudo-base) [19]. Studies have shown that at a mild pH value (3.5), the thermal degradation of anthocyanins takes place through the open chalcone form, therefore, a temperature increase favors degradation [19]. This is in agreement with our findings, which showed higher degradation degrees with an increasing storage temperature of the anthocyanin solutions.

For the light test, it was difficult to formulate a mechanism. In the existing literature, different data have been reported regarding the degradation kinetics of ANTH under light irradiation. Modesto Junior et al. studied the light (fluorescent, incandescent, and ultraviolet) degradation of ethanolic anthocyanins in grumixama extracts and fitted the Weibull model [30]. Chisté et al. reported first-rate degradation kinetics for ANTH from mangosteen peel under different light irradiation (fluorescent, incandescent, ultraviolet, and infrared lights) [55]. Different half-lives were reported by the authors of these studies and the explanation was that the ANTH composition varied when passing from one species to the other as well as the presence of other species in the plant environment [30]. Regarding the mechanism of decomposition under light, the type of light irradiation is a key factor to be considered. Modesto Junior et al. reported an increased susceptibility of ANTH to degradation under UV light irradiation when compared with fluorescent and incandescent light [30]. Under UV light, photons are absorbed in conjugated double bonds of aromatic compounds, reducing their stability [30,56]. Radical species might be involved in this case, and the degradation might undergo a different mechanism compared with the mechanism presented above (Scheme 3).

Identifying the degradation products of blueberry anthocyanins was not the purpose of our study, but we can assume that their degradation involves the participation of water and additional oxidative degradation processes autocatalyzed by organic acids as citric, malic, oxalic, succinic, and lactic (that can be found in blueberries) coupled with bond cleavages [19,57,58,59]. The literature data indicate that the degradation of anthocyanins in water-containing media at acidic pH follows the pathway of flavylium cation hydration- carbinol pseudo-base—chalcone, followed by sugar loss and the degradation of various anthocyanidin chalcones through bond cleavage. For the blueberry extracts, taking into account the presence of delphinidin, petunidin, and malvidin as the main anthocyanidins, the main compounds resulting from BBE ANTH decomposition are probably phloroglucinaldehyde, gallic acid, and its mono- and di-methoxy derivatives: 3,4-dihydroxy-5-methoxybenzoic acid and 4-hydroxy-3,5-dimethoxybenzoic acid (syringic acid) [19,58,59]. These compounds polymerize into brown compounds in reaction with each other, with other phenolic compounds, or with non-phenolic compounds present in the mixture (amino acids, etc.) [57]. In Figure 4, the presence of polymeric brown compounds could be observed in sample (4)—left image. In this sample, the degradation degree was the most elevated compared with the other samples (97%), allowing these compounds to be observed. Polymeric compounds also probably formed in the rest of the samples, but to a lesser degree, and the presence of remaining anthocyanins screened their observation.

Therefore, the anthocyanin decomposition products that we expected to be formed in the studied BBE extracts are supposed to be non-toxic compounds, similar to those formed through the decomposition of anthocyanins in plant tissues. Regarding the effects on human health, simple phenolic compounds (like gallic acid and its mono- and di-methoxy derivatives) also possess anti-inflammatory and antioxidant activities [60]. The antioxidant activity of the phenolic compounds that result from ANTH decomposition is expected to be less potent when compared with the antioxidant activity of ANTH. This assumption was confirmed by a previous study investigating the effects of the thermal degradation of ANTH on the antioxidant activity of vegetal samples. The study revealed that the samples resulting from thermal degradation, containing phenolic acids and phloroglucinaldehyde as terminal degradation products, presented decreased antioxidant activity, concluding that the phenolic compounds obtained after heating could not compensate for the loss of the antioxidant activity of ANTH from the initial samples [61]. Therefore, we assumed that, following degradation, the studied BBE extracts would contain non-toxic compounds possessing decreased antioxidant activity compared with the initial extracts.

#### Precautions Imposed on the Study

The study of the degradation/decomposition processes of active components from natural extracts requires certain clarifications and precautions in the interpretation of the experimental data.

1. Natural fruit extracts are multicomponent systems whose properties and characteristics depend on the quality of the fruits, namely: (i) the ripeness level inducing the color intensity; (ii) the freshness reflecting the hydration/dehydration degree; (iii) the provenance related to the plant cultivation place, which significantly affects the concentration of the active components; and (iv) the treatment method to which the respective fruits are subjected can generate the appearance of other compounds sensitive to the extraction process (e.g., from pesticides or from various treatments applied to preserve freshness), thus determining a change in composition. All of the above can lead to obtaining non-reproducible, but relatively close results, between studies. Although natural extracts do not offer the accuracy of results obtained in simulated laboratory media where certain interactions between different compounds can be controlled, these are of particular interest, revealing the evolution of the chemical species into a real complex environment.
2. The investigation of chemical compound decomposition processes can be carried out in controlled laboratory conditions or natural conditions close to the real ones during handling/storage.
3. The results obtained in controlled laboratory conditions depend on the investigating parameters such as the isothermal regime, the luminous flux emitted by a source, related to the unit angle in which it emits, the spectrum of UV rays (UV-A, B, C, or extreme UV), the wavelength of microwaves (Hertzian waves), the current density or potential in electrochemistry, etc. Thus, the application of these parameters, in synergism or individually, can lead to slightly divergent results and conclusions, but which certainly present important indications in the development of degradation processes in a common/natural/real environment.
4. Investigations in common environments, at operating parameters involving room temperature and natural light, can provide results supplementing the information reported by those conducted under controlled laboratory conditions.
5. These research studies do not foresee additional risks compared with those generally invoked for natural extracts and do not assume an ephemeral acceptance. Natural extracts studied under conditions close to reality can constitute starting points for investigations in more complex environments, such as the human body, where a multitude of biochemical and metabolic factors intervene, which can generate unexpected and surprising responses and results, different from those provided by simulated/natural controlled/uncontrolled environments.
6. Finally, based on the above, the comparison between the results reported by different studies is sometimes deficient, but these can fall within a certain error margin involving the environmental factors and preparation of extracts.

## 4. Conclusions

ANTHs are important dietary components with antioxidant properties.

In this study, ethanol/water extracts of different fresh fruits were obtained, and the ANTH content was determined. The TAC content in the analyzed fruits was between 256 and 12 mg/100 g fresh fruit, decreasing as follows: blackcurrants > blackberries > blueberries > raspberries > strawberries > plums.

The degradation of BBE ANTHs in four experimental conditions was investigated and revealed that ANTH stability decreased as follows: sunlight test << shadow test ≈ dark test < cold test. The best conditions for extract storage were in the absence of light and at low temperature (cold test), when a low percentage of color removal (2.4%) was achieved after 10 days. The storage conditions most detrimental to the conservation of BBE ANTHs were in direct sunlight, conditions that caused a gradual and almost complete degradation of ANTHs, reaching a value of 97.4% color removal after 10 days of exposure. The other two storage conditions, at room temperature (shadow and dark test), indicated similar results, with a slightly more elevated stability in the complete absence of light (dark storage). Both sunlight and temperature exposure had a negative impact on ANTH stability in alcohol solutions. Therefore, BBE extracts should be handled carefully, avoiding storage in inappropriate conditions (light and temperature exposure) to maintain their qualities.

A kinetic study was realized, indicating that ANTH degradation proceeded according to zero-order kinetics in the case of blueberry extract exposure to sunlight, and respected pseudo-first-order kinetics for the other storage conditions (shadow, dark, and cold).

The findings of this study bring new information to the field of the storage of anthocyanin-containing alcoholic extracts, with applicability in the packaging and storage of alcoholic extracts (beverages, essences, essential oils). Colorful ANTH rich extracts may be used in the food industry as nutraceuticals due to their health promoting properties, among which the antioxidant properties are outstanding.

## Figures and Tables

**Table 1:** Anthocyanin concentration (AC) in the extract solutions, total anthocyanin content in fruits (TAC) compared with the TAC content reported in the literature.

Fruit	AC ^a^ in Extract Mean ± RSD ^c^ (mg/L)	TAC ^b^ Content Found in This Study Mean ± RSD ^c^ (mg/100 g Fresh Fruit)	TAC ^b^ Content Reported in the Literature (mg/100 g Fresh Fruit)	Literature Reference
Blackcurrant	431.14 ± 7.09	255.49 ± 7.09	130–476	[3,41,42,43]
Blackberry	195.19 ± 3.26	112.88 ± 3.26	82–325	[3,41,43,44]
Blueberry	138.18 ± 7.50	80.74 ± 7.50	25–495	[3,41,45,46]
Raspberry	54.11 ± 3.21	31.37 ± 3.21	20–687	[3,41,43]
Strawberry	43.05 ± 10.90	25.66 ± 2.80	19–55	[3,41]
Plums	20.04 ± 11.04	11.81 ± 11.40	5–57	[47]

^a^ AC: Anthocyanin concentration in the extract solutions, expressed as cyanidin-3-glucoside (cyd-3-glu) equivalents (mg/L), were calculated using Equation (1); ^b^ TAC: Total anthocyanin content in fruits, expressed as cyanidin-3-glucoside (cyd-3-glu) equivalents (mg/100 g fresh fruit), was calculated with Equation (5). ^c^ RSD: Relative standard deviation, calculated as: RSD = SD × 100/Mean.

**Table 2:** The absorbance variation over time with respect to the environmental storage conditions.

Time (Days)	Absorbance ± SD			
	Cold Test	Shadow Test	Dark Test	Sunlight Test
0	0.8829 ± 0.0007	0.8829 ± 0.0007	0.8829 ± 0.0007	0.8829 ± 0.0007
2	0.8782 ± 0.0240	0.8680 ± 0.0242	0.8589 ± 0.0118	0.7028 ± 0.0169
4	0.8725 ± 0.0150	0.8447 ± 0.005	0.8402 ± 0.0089	0.5040 ± 0.0113
6	0.8657 ± 0.0388	0.8290 ± 0.009	0.8222 ± 0.0214	0.2386 ± 0.0070
8	0.8656 ± 0.0489	0.8077 ± 0.0081	0.8024 ± 0.0138	0.1032 ± 0.0020
10	0.8616 ± 0.0284	0.7829 ± 0.0092	0.7785 ± 0.0103	0.0228 ± 0.0060

**Table 3:** Kinetic parameters obtained for the ANTH degradation reaction comparatively presented from the zero-order and first-order reaction kinetics.

Sample	Zero-Order Reaction					
	Initial Absorbance (A_o_) Values			R^2^	k/days^−1^	t_1/2_/days
	Experimental Value		Calculated Value			
Sunlight test	0.8829		0.8637	0.9782	0.0909	4.86
Shadow test	0.8829		0.8879	0.9782	0.0108	40.87
Dark test	0.8829		0.8815	0.9976	0.0101	43.61
Cold test	0.8829		0.8819	0.9555	0.0022	200.66
**First-Order Reaction**						
**Sample**	**Initial Absorbance (A_o_) Values**		**R^2^**		**k/days^−1^**	**t_1/2_/days**
	**Experimental**	**Calculated**	**From Equation (12)**	**From Equation (11)**		
Sunlight test	0.8829	1.3898	0.8571	0.9092	0.354	1.96
Shadow test	0.8829	0.8865	0.9926	0.9917	0.012	57.76
Dark test	0.8829	0.884	0.9891	0.9872	0.013	53.32
Cold test	0.8829	0.8819	0.9568	0.9568	0.0025	277.26

**Table 4:** Reaction-order and kinetic data for the ANTH degradation reaction under different laboratory conditions.

Sample	Applied Kinetic Model	k/days^−1^	t_1/2_/days
Sunlight test	Zero-order reaction kinetics	0.0909	4.86
Shadow test	Pseudo-first-order reaction kinetics	0.012	57.76
Dark test	Pseudo-first-order reaction kinetics	0.013	53.32
Cold test	Pseudo-first-order reaction kinetics	0.0025	277.26

## Data Availability

All of the data are available in the main text of the article. More details can be provided on request.
